# A Treatise on the Venereal Disease

**Published:** 1783

**Authors:** 


IV. *A Treatise on the Venereal Disease. By* G. Renny, *surgeon to the Athol Highlanders.* 8vo. Murray, *London*, 1782. 171 pages. 3s.

IN a short introduction to this work the author offers a few remarks on the cause on which the diversity of treatment of the venereal disease, in its simple and confirmed state, is founded. Without pretending to determine whether the gonorrhœa and confirmed lues are the effects of the same virus, he thinks that as it is certain that in ninety-nine cases of a hundred a gonorrhœa is not succeeded by a general lues, this is a sufficient authority for the practitioner to regulate the cure upon different principles.

The work itself is divided into nine chapters. In the first chapter Mr. Renny treats of the gonorrhœa. This he considers as an effect of the inflammation excited in the mucous glands of the urethra, by the venereal poison: The disease, therefore, he thinks is purely local, and accordingly, in the cure he insists chiefly on the use of the following injection, which is to be retained for a short space in the urethra, and applied to every part of the internal membrane by rubbing the finger along the course of that tube.

℞ Calomel. pp. gr. v.

Sacch. Saturn. ℈j. Pulv.

Pulv. e Ceruff. Comp. ʒj.

G. Arabic ℥ſs. ſolv. in aq. font. bull. ℥viij.

M. f. inj.

During the uſe of this remedy, and while the diſeaſe continues, the teſticles are to be carefully ſuſpended, and the patient is directed to abſtain from exerciſe and inflammatory diet. If the ſymptoms of inflammation run high, the uſe of the injection is to be ſuſpended till they have been removed by venæſection and a gentle purgative.

Mr. Renny cloſes his account of the gonorrhœa with ſome remarks on gleets. The cauſes of theſe, he is of opinion, may be referred to warts in the urethra, to topical irritation unconnected with obſtruction, or to a relaxed ſtate of the mucous glands of the urethra, and a debility of the ſyſtem in general. Of all theſe cauſes, the latter, he obſerves, is the moſt frequent, being very uſually an attendant on gonorrhœas, which are treated by the antiphlogiſtic method, without the uſe of injection.

In the ſecond chapter Mr. Renny treats of inflammation of the teſtis, a ſymptom, he obſerves, which is ſo far from being confined to thoſe caſes where injections are employed in the cure, that it occurs oftener where the antiphlogiſtic method method has been purſued, and nothing aſtringent applied, ſeeming to be the cauſe rather than the effect of a ſudden ſtoppage of the diſcharge.

This complaint, we are told, ſeldom occurs where the teſticles are carefully ſuſpended. In proof of this the author informs us, that ſoon after his appointment to the ſurgeoncy of a highland battalion, four out of eight ſoldiers, reported with gonorrhœas, were attacked with inflammation of the teſtis. This he attributed to their wearing no breeches, and their omitting the uſe of a bag truſs. Since that time, although he has had occaſion to treat an hundred caſes of gonorrhœas in the ſame battalion, yet by taking care to have the teſticles ſuſpended, an hernia humoralis has been uniformly prevented.

The mode of treatment recommended, when this ſymptom has actually taken place, conſiſts in general bleeding, the application of leeches to the ſcrotum, the uſe of warm fomentations (in preference to poultices, which are ſaid to do harm by their weight) and vomits. Mr. Renny prefers clyſters to purgatives as a means of obviating coſtiveneſs in theſe caſes, becauſe purgatives are often productive of inconvenience and pain to the patient by obliging him to alter the the poſture of his body when going to ſtool, and this he thinks more than balances for any good they may do as evacuants.

In the next chapter the author ſpeaks of chancre. He is of opinion, that every ulcer of this ſort is produced by the ſame cauſe operating originally at the time of infection, and not by the diſcharge from a particular one begetting a ſecond, and ſo on. This, he thinks, is rendered probable by their number being always determinate early, and each ulceration preſerving afterwards its own limits, whereas if this was not the caſe, we might expect to ſee them multiplied *ad infinitum*, as in the progreſs of the complaint there are few ſpots, either on the glans or prepuce, that are not expoſed to the application of the venereal matter. Mr. Renny, conſidering a chancre as an effect of the venereal virus lodging on the part during coition, allows that in ſome caſes it may be local; but as in every ulceration there is a ſtrong preſumption that an abſorption may have begun, he thinks we have ſufficient grounds for conſidering chancres as a complaint in which the habit is almoſt certainly affected, and to conduct the cure upon ſuch principles. In conformity to theſe ideas he contents himſelf with keeping the ulcer clean, and dreſſing dreffing it with fimple digeftive ointment, and for the cure relies chiefly on mercurial frictions.

The fubject of the fourth chapter is phymofis. The author divides this into two fpecies. The firft fpecies we are told is occafioned by chancres, and diftinguifhed by the hardnefs and thickened ftate of the prepuce, and the difcharge from the part. In the fecond fpecies, the complaint may be often traced from expofure to cold, and the inflammation, though in general confiderable, is not attended with hardnefs of the prepuce, but only feems very red, with a gloffy chryftalline appearance. In both of thefe fpecies, Mr. Renny thinks mercurials have a bad effect, whether applied topically or to the fyftem at large. In the cure he recommends the antiphlogiftic method, and the frequent application of warm milk and water to the part. Where the inflammation is independent of chancres, he employs a folution of faccharum faturni as a fomentation, and as the difeafe is here purely local, he thinks it may in general be cured without the ufe of mercury. He remarks likewife that in the phymofis from chancres the inguinal glands are for the moft part fwelled, whereas in the more fimple fpecies this fymptom takes place more rarely, and where it does occur

ſeems to be merely the effect of ſympathy, as it conſtantly keeps pace and diſappears with the topical inflammation.

Mr. Renny proceeds next to paraphymoſis. Although this ſymptom may ſometimes be the effect of chancres, yet he is of opinion that in the generality of caſes it is occaſioned by an inflammation excited in the glans and prepuce, in patients who have naturally a difficulty in denuding the penis. There are few caſes, he obſerves, in which a timely ſcarification will do more good, or where delay will be productive of more miſchief, than in this. The ſurgeon is therefore directed immediately to make an inciſion on each ſide of the penis, deep enough to remove the ſtricture effectually, and then to finiſh the cure by proper fomentations and the antiphlogiſtic courſe. A few days after the operation, we are told, a conſiderable degree of ulceration comes on between the ſides of the foſſa made by the ſtricture. This, which is always a troubleſome ſymptom, has often been improperly conſidered and treated as a mercurial ſore. If the inflammation has advanced ſo rapidly as to ſhew every mark of an approaching gangrene before the ſurgeon is called in, he is adviſed not to attempt the operation, but to have recourſe to to the bark and opium, which are ſaid to have produced a wonderful good effect, even in caſes that ſeemed deſperate.

In the ſixth chapter the author treats of bubo. A tumour of this ſort, he thinks, ought to be repelled by every means in our power: at the ſame time, however, he confeſſes that the practice of bringing buboes to a ſuppuration is in general more ſucceſsful, as a caſe ſeldom occurs where a bubo ſuppurates without ending in a complete cure, and he has very often ſeen relapſes attendant on their repulſion. This difference he attributes to the difficulty with which patients are induced to continue the uſe of mercury after the tumour is diſperſed and they think themſelves well, whereas ſo long as any ſore remains in the groin they readily ſubmit to whatever is preſcribed.

In the opening of tumours of this ſort Mr. Renny prefers inciſion to every other method, and thinks it moſt adviſeable, in general, to remove a circular piece from the forepart of the bubo with a common biſtoury. If the ſore ſpreads, and the diſcharge is conſiderable, recourſe is to be had to bark and opiates till the ſymptoms are more favourable to the exhibition of mercury.

Towards the cloſe of this chapter Mr. Renny offers a few remarks on warts. Theſe he divides into venereal and topical. The former, he obſerves, diſappear with the other ſymptoms. The latter are to be removed by cauſtic or the ſciſſars, and their return prevented by mixing a ſmall portion of the powder of ſavine with the dreſſings.

The next chapter Mr. Renny allots to what he calls confirmed ſymptoms, a mode of expreſſion, as he tells us, which he adopts rather out of reſpect to the univerſal opinion, than from any conviction that the particular ſymptoms which remain ſtill to be treated of deſerve to be ſeparated from chancre and bubo. He firſt ſpeaks of ulcerations of the fauces; theſe he conſiders as certain marks of a confirmed lues, and he recommends the ſame treatment here as in chancres. Excreſcences round the anus and on the ſcrotum require a management ſimilar to warts. Venereal ophthalmia is to be obviated by general and local evacuations, by the antiphlogiſtic regimen, and the cure finiſhed with mercury. Ulcerations on the ſurface of the body, pains in the head and limbs, diſeaſes of the bones, deafneſs, &c. are ſo complicated with particular conſtitutions and occur ſo ſeldom in pracpractice of late years, on account of the cure being so much better understood, that he declines offering any thing particular on these heads.

The eighth chapter contains a few observations on the preparations of mercury. These are divided by our author into the acrid and mild. Those of the latter class, he thinks, are in the end as efficacious as the others, and much easier to manage. He prefers mercurial ointment to every other preparation, but if the patient refuses to submit to this, the common mercurial pill, triturated with honey or mucilage instead of turpentine, may be substituted.

In the last chapter of the work the author treats of the action of mercury. He is of opinion that in proportion to the more uniform effect produced by this mineral upon every secretion, and the less tendency it has to run off by a particular one, so will it more essentially serve the curative purpose it was designed for; and upon this principle, that the increase of any one secretion, naturally produces a diminution of every other. Hence he is equally averse to purging and to salivation.

He points out the necessity of attending to the support of the system during the exhibition of mermercury, a proper ftrength of circulation feeming to be peculiarly neceffary to enable it to expel the venereal virus completely. Laftly he inculcates the neceffity there is for continuing the adminiftration of mercury a certain time after the fymptoms have difappeared.

So far the treatment of the difeafe has been confidered as applicable to the male fex. In females, we are told, the cure will be infinitely more fimple, as moft of the topical fymptoms are wanting, and will only differ from the other, in proportion to the delicacy of the female conftitution, which will require a greater attention in the exhibition of mercurials.

The work clofes with a relation of fix cafes, illuftrative of the doctrines delivered in the preceding chapters.

